# Improving the EFMs quality by augmenting their representativeness in LP methods

**DOI:** 10.1186/s12918-018-0619-1

**Published:** 2018-11-20

**Authors:** José F. Hidalgo, Jose A. Egea, Francisco Guil, José M. García

**Affiliations:** 1 Grupo de Arquitectura y Computación Paralela, Universidad de Murcia, Murcia, Spain; 2 Dpto. de Matemática Aplicada y Estadística Universidad Politécnica de Cartagena, Cartagena, Spain

**Keywords:** Metabolic networks, Pathways and EFMs, Representativeness and quality, Flux modes, Linear programming, Systems biology

## Abstract

**Background:**

Although cellular metabolism has been widely studied, its fully comprehension is still a challenge. A main tool for this study is the analysis of meaningful pieces of knowledge called modes and, in particular, specially interesting classes of modes such as pathways and Elementary Flux Modes (EFMs). Its study often has to deal with issues such as the appearance of infeasibilities or the difficulty of finding representative enough sets of modes that are free of repetitions. Mode extraction methods usually incorporate strategies devoted to mitigate this phenomena but they still get a high ratio of repetitions in the set of solutions.

**Results:**

This paper presents a proposal to improve the representativeness of the full set of metabolic reactions in the set of computed modes by penalizing the eventual high frequency of occurrence of some reactions during the extraction. This strategy can be applied to any linear programming based extraction existent method.

**Conclusions:**

Our strategy enhances the quality of a set of extracted EFMs favouring the presence of every reaction in it and improving the efficiency by mitigating the occurrence of repeated solutions. The new proposed strategy can complement other EFMs extraction methods based on linear programming. The obtained solutions are more likely to be diverse using less computing effort and improving the efficiency of the extraction.

## Background

Stoichiometry can be used to construct a model of the biological system inside a cell. The resulting model is a genome-scale metabolic network (GSMN) and it explains how the chemical reactions occur and how the metabolites are produced and consumed during the metabolic process. Building high-quality GSMNs must follow some steps [1]. The model can be enriched with *omic* data. Constraint-based mathematical techniques are useful tools to analyse GSMN models. However, automatic characterisation of the biochemical reactions present in a particular metabolism constitutes a challenge [2].

GSMN can also be seen as the addition of interrelated sub-networks. To support life, each sub-part of the metabolic network has to accomplish its specific biological function. Pathways are a special class of sub-networks. They are steady-state and thermodynamically feasible subsets of reactions, that means that mass balance remains unaltered while the reactions produce and consume metabolites. Elementary flux modes (EFMs) [3] are non-decomposable pathways. In other words, eliminating any reaction from an EFM would result in an infeasible pathway from the thermodynamical point of view. Since the EFM concept was introduced, different mathematical and computational procedures to find all (or some of) the EFMs from GSMNs have arisen. The nature of the EFM concept suggests that optimization techniques could be applied as extraction tool. The pretended solution of each run of an optimization technique would be an EFM.

A very well-known optimization technique is Linear Programming (LP). There are very efficient implementations of linear program solvers available [4, 5]. Those solvers perform similarly and are deterministic. In order to obtain different solutions while solving a linear program, the program must be additionally constrained with different sets of conditions. Stoichiometric equations also express more or less evident dependency relationships between reactions and metabolites along the network. Previous studies approximate the computation of flux coupling [6, 7] and metabolic coupling [8] discovering those hidden relationships across the metabolic network. It is important to deal with the side effects of coupling relationships because they can condition the solver to get the same solution even if the constraints explicitly included in the linear programs are different or even strictly disjoint.

The length of the modes and the presence of every metabolic reaction in the set of solutions have been studied on [9]. This paper presents a new strategy to improve existent LP-based extraction methods of pathways and EFMs reducing the appearance of some recurrent reactions in the solutions and, at the same time, increasing the probability that every considered reaction appears in the set of extracted EFMs, which would enhance the diversity of such set. This would improve the representativeness of this set of by promoting the occurrence of reactions from all over the network. The implementation of the idea exposed above has a straightforward applicability when using extraction methods based on linear programming.

There are two main ways of getting different solutions (at least, to reduce the proportion of repeated ones): find a method to generate seeds that produce different solutions or modify the objective function so that the optimization process tends to get solutions that are different from the ones previous obtained. In this paper, we are interested in studying the viability of this second approach.

In order to drive the LP solver to different solutions on each run, in this paper it is proposed to dynamically modify the objective function to coerce the LP solver to consider less used reactions. As it seems obvious that proposing the most used reactions to be part of the seed once and again favours the occurrence of repeated solutions, a plausible method to try to avoid repetitions in the new solution is to penalize any reaction that has been included in previously obtained solutions. Our proposal can be viewed as a mechanism to help LP solvers not to fall insistently in local minimums.

The paper is structured as follows. In the “Methods” section, an introduction on the the use of linear programming for EFM extraction as well as comments on the implementation and the issues found using this approach are provided. The “Results and discussion” section presents the results of our approach for some study cases, discussing the tradeoffs involved in the modification of the optimization problem for each iteration. The final Section provides some conclusions and ideas for further investigation.

## Methods

### The problem of pathways and EFM extraction

Network metabolic models let a full or a context-specific analysis of the role that plays any reaction or metabolite inside the cell. It is particularly interesting the role that they play in particular disease research, where is needed to extract a specific piece of information from the full network. Pathways and Elementary Flux Modes (EFMs) are types of sub-network whose analysis have been remarked by plenty of works [10, 11]. The main drawbacks of using EFMs are the high computational cost to enumerate them and, when obtaining a subset of all the possible EFMs in a GSMN, the uncertainty in having enough biological relevance. There are different proposals to enumerate subsets of EFMs in GSMNs [12–15]. There are also a family of algorithms for context-specific metabolic network reconstruction that ensuring the presence of a key set of reactions within the simplified resulting model.

There are two main groups of computational approaches to extract information from metabolic pathways: path-finding and stoichiometric [16]. The first ones consider the network as a directed graph and explore it [17–19]. The main disadvantage is that they do not use stoichiometric coefficients during the exploration process. The second ones use the stoichiometric data to do calculations during the process. Linear Programming and Null-Space Algorithm [20] are some of the mathematical strategies applied to find pathways, mainly solving the system of linear equations proposed by the stoichiometric matrix.

### Genomic metabolic networks as a system of equations

Each metabolic reaction inside a cell can be represented by its correspondent stoichiometric equation. All the equations are arranged in a stoichiometric matrix where columns represent metabolic reactions and rows represent metabolites. The matrix values represent the stoichiometric coefficients for the production or consumption of metabolites on each reaction.

Be *S* a stoichiometric matrix (i.e., the matrix of coefficients of the biochemical reactions for the studied network). These coefficients represent the frequency at which reactions occur at the steady-state or, equivalently, the rate of metabolites production/consumption through the reactions. A feasible sequence of reactions occurring inside a cell can be comprised in a vector called flux rate that contains the reaction rates, that is, the values for the variables of the system of equations represented in the *S* matrix. If *R* is the full set of metabolic reactions, the flux vector $\vec {v}$ must fulfil the steady-state condition (Eq. 1) and the thermodynamic constraints (Eq. 2).

1 $$\begin{array}{*{20}l} S \cdot \vec{v} = \vec{0}  \end{array} $$

2 $$\begin{array}{*{20}l} v_{r} \geq 0, \quad \forall{r}\in R  \end{array} $$

Equation 1 (i.e, steady-state condition) involves a balance among all metabolites and constant concentrations. The thermodynamic constraint forces each irreversible reaction present in the solution to have a positive rate. This is a biological thermodynamic restriction. Many methods need to split reversible stoichiometric reactions into two irreversible reactions to implement the accomplishment of the thermodynamic restriction. This are a very common strategy and it is needed when using linear programming as a mathematical tool.

Once the above equations are solved at least two different solutions are obtained: the trivial one and other that represents the whole network. A pathway is just a solution of the solutions: a vector flux that is positive and verifies the steady-state condition. It can be viewed that any solution is associated to a subset of the full set of reactions formed by those that has non-zero rate. Starting from the whole network, and working in an iterative way, the goal is to find subsets of reactions, the ones associated with pathways, that correspond to solutions of the system. So, a pathway is a subset of the full set of reactions satisfying that we can find a flux-vector that is a solution of the previous equations and whose support (its non-zero values) are exactly the values corresponding to reactions in the pathway. If the metabolic network is represented as a graph, a pathway can be seen as a sub-graph.

It is said that $\vec {v}$ is the vectorized representation of an EFM if it is not decomposable (i.e., it can not be written as a positive linear combination of flux rate vectors representing any other pathway in the network). It is well-known that a pathway $\overrightarrow {v}$ is an EFM if and only if there is no other pathway whose support is strictly included in that of $\overrightarrow {v}$. Non-decomposability, also called *minimality*, is the condition that let transform the extraction method into an optimization problem instead of just a system of equations.

The biological relevance of an EFM is inherited from the fact of the uniqueness of the set of EFMs and the canonical quality as a set of vectors that can generate any pathway, even those unobserved [11, 21].

### Linear programming

Linear programming (LP) is the most popular optimisation-based approach for EFMs extraction. LP is being widely used to reduce the complexity of combinational problems in systems biology introducing optimization objectives that lead the search and constraint the space of solutions to a subset within an specific focus [22–24].

The existing literature describes how to formulate an EFMs extraction problem as a linear program considering the constraints defined in the previous Section [13] (Eq. 3).

3 $$\begin{array}{*{20}l} \text{Minimize} \quad & \sum\limits_{i=1}^{n} v_{i} \\ \text{subject to} \quad & S \cdot \vec{v} = \vec{0}  \\ & v_{r_{i}} \geq 0 \quad \forall r_{i} \in R  \end{array} $$

Once a linear program is obtained, it can be solved using, for instance, the Simplex Algorithm implemented in plenty of LP solvers.

A trivial solution for the linear program posed in Eq. 3 is $ v_{r_{i}} = 0 \quad \forall r_{i} \in R$, which provides no biological information. Therefore, we must impose different conditions to modify the linear program by adding new constraints to the LP problem to obtain different solutions from the trivial one.

Additional constraints can be seen as set of reactions forced to have positive fluxes (positive constraints) or, contrarily, as set of reactions forced to be inactive, which means that their associated fluxes are equal to zero (negative constraints).

We define a *seed* as a constraint of this kind in reference to the fact that a seed is the precursor of one solution. Seeds are needed to coerce the solvers to find non trivial, thermodynamically feasible solutions and can also be used to try to find solutions that are different from the previous ones. There are existing heuristics that, searching for good seeds, introduce jumps as a strategy to escape of the attraction of local minimums. The attraction of those local minimums is one of the causes of the repetitions in the solutions found by the LP solver.

### EFM extraction using LP

Observe that the objective function $\text {Min}\sum \limits _{i=1}^{n} v_{i}$ was introduced in order to transform our system of equations into an optimization problem. Intuitively, this function reaches its minimum when the number of non-zero addends is minimal and so we expect to get solutions that are EFMs. But it is easy to modify the proposed function to obtain a whole set of functions with the same behaviour (we can use, for example, $f(\overrightarrow {v})=\sum \limits _{i=1}^{n} \lambda _{i}v_{i}$ for any set of positive numbers *λ*_*i*_).

A fairly approach for the extraction of EFMs is to use a computer program based on linear programming. This program essentially consists of a number of iterations of the Simplex method. Algorithm 1 shows the typical composition the program used. The main loop is iterated at least as many times as solutions needed (*N*).


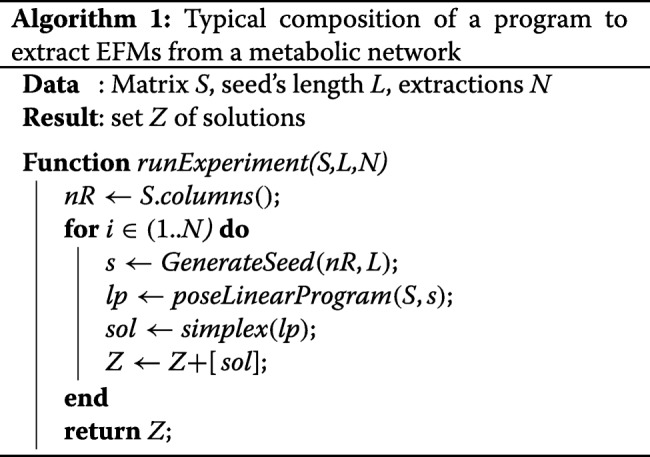


A seed is an input for the LP solver that is computed in the constraints Section. It can be said that a linear programming based extraction method is basically a strategy to produce relevant seeds.

### Seed generation: infeasibilities

There are two different issues that can appear when using seeds. Firstly, different seeds can produce exactly the same solution (as it is well-known). So, it is not enough to produce a large set of different seeds to obtain a large set of different pathways. It is not easy to choose seeds to get new pathways, and the difficulty increases as more solutions are computed.

On the other hand, the chosen seed can led to a problem that has no solution at all. That is, when a certain set of reactions are imposed to be in the solution and another set cannot appear, sometimes contradictory conditions are being imposed. Suppose, for example, that a reaction *r*_1_ produces a metabolite *m* and it can only be eliminated by another reaction *r*_2_. If the reaction *r*_1_ is imposed to be part of the solution but not the reaction *r*_2_, it is impossible to get a solution satisfying the steady-state condition (*m* will be produced and cannot be eliminated). A seed is called infeasible if its associated linear-program problem has no solution.

In order to avoid infeasibilities, the set of seeds have to be restricted to a positive ones, that is, seeds that only determine what reactions are required to be part of the solution but not what are forbidden to be.

### Seed generation: repetitions and representativeness

Another frequent problem of a set of solutions after run an long extraction experiment is the under or over-representation of some part of the metabolic networks. This phenomenon comes associated to some kind of affinity of the seed generator or a lack of dispersion or randomness of its conception.

Each iteration requires its own seed that is incorporated as a part of the final computed solution. The process of building a seed consists of choosing what reactions are included in the constraint and, therefore, in the particular solution of a constrained LP using that seed. Defining different constraints from previous ones does not guarantee different solutions. As the Simplex algorithm is deterministic, the method to compose the seeds is critical to get a significant set of solutions.

Due to the fact that the seed induces the final solution, the correspondent seed generator is also responsible of the quality of the final set of solutions. It seems clear that the generation of a new seed should be influenced by the previous ones, and in that way, avoid to obtain the same EFM twice. For example, graph-affinity based approaches use the graph adjacency of reactions to build seeds, so in some way assures some kind of minimality by forcing the connectivity of the solution. The counterpart is that the adjacency can cause over-representation of a determined subgraph in detriment of the global meaning. It is also worth to say that sometimes the same problems are due to the exhaustion of the method to generateb different seeds.

A completely random seed generator can be considered as the simplest and fair seed generator. It could be a module of an invented extraction method that produces constraints for a linear program on every iteration. This is a neutral way to generate seeds that frees us to record previously produced seeds to avoid repeated seeds. Algorithm 2 shows how it works.


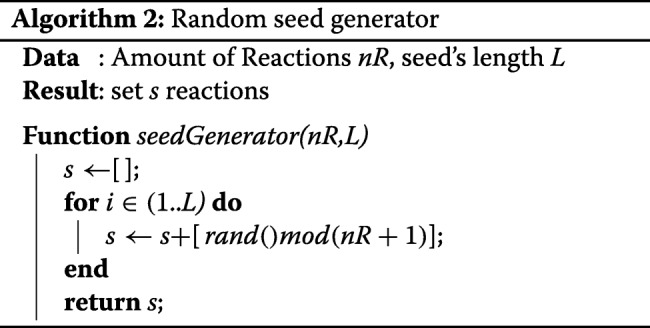


### Frequencies

**Fig. 1:**
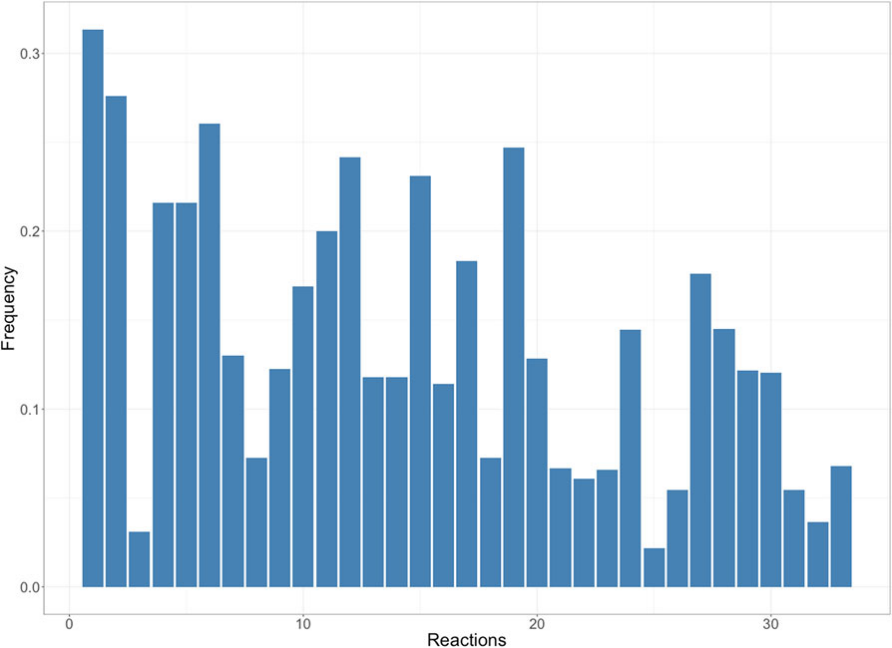
Bargraph frequency/reaction. Bar graph for the frequencies of an experiment. Each bar corresponds to the frequency (y-axis) of one reaction. The order for the reactions in the x-axis is irrelevant but it is the same through all the experiments

Pathways and EFMs extraction methods incorporate strategies to avoid repeated solutions as much as possible. The frequency distribution of the reactions present in the solution is a characteristic of each extraction method. Be *N* the number of iterations performed in one optimization problem and *O*_*i*_ the number of occurrences of the reaction *r*_*i*_ in the experiment. The frequency of occurrence for each *r*_*i*_ is $F_{i} = \frac {O_{i}}{N}$. A typical frequency distribution that outcomes for one experiment is shown in Fig. 1.

The frequency distribution is somehow conditioned by the extraction method, but there are other characteristics that can influence on it. For example, the intrinsic properties of the biological network (its size or its coupling relations), or even the amount of runs done for an experiment can influence the outcome.

It is reasonable to expect similarity between two sets of frequencies obtained in two separated experiments. So, if the frequencies are almost the same, we can expect that they express properties of the network and not of the chosen extraction method. This can open a new path to understand why some reactions appear more often in the set of solutions than others.

### Penalizing the objective function

Depending on structural properties of the network or magnitude factor of the stoichiometric coefficients, solvers could tend to include the same reactions in the final solution in almost any case, and this can lead to obtain repeated solutions independently of the chosen seed.

In this work, a *penalization* scheme that modifies previous results is proposed in order to increase the number of different solutions, and/or to get them faster. Our aim is to force the solver to avoid (when possible) reactions that are present in solutions computed previously in order to get new ones.

The proposed strategy consists of modifying the objective function with sets of positive *weights* {*w*_*i*_} to formulate it as ${\sum \nolimits }_{i=1}^{n} w_{i}\cdot v_{i} $ instead of ${\sum \nolimits }_{i=1}^{n} v_{i} $. These weights may vary depending of the number of previously computed solutions containing any reaction so that overrepresented reactions get a higher weight. Due to the fact that we are minimizing the function, the solver will tend to avoid reactions with higher weights if possible.

Observe that this strategy is not equivalent to the use of negative seeds. The latter one can lead us to infeasibilities while the use of weights only discourages the use of reactions that has appeared before but does not ban them. Therefore, different sets of weights may provide different solutions even when using the same seed.

It is interesting to study the influence of a set of weights over the solutions. To that, the first step is to analyse if the use of weights has a real impact on the set of solutions obtained. Once we are sure that our approach modifies the set of solutions, a second question is how to choose the weights in order to obtain sets of solutions with the desired properties: the number of repeated solutions should be as small as possible and the set should be representative (as many different solutions as possible).

To answer these questions we have to compare the outcomes of different experiments by applying different statistical tools to compare the results. In this work, we have used the Wilcoxon signed-rank test [25] and a test based on *chi-square*.

Initially, all the objective function weights equal 1 (i.e., Eq. 3). We aim to modify some of these weights to obtain different solutions from the LP. In particular, we increase the weights of the fluxes associated to the reactions with higher frequencies in the set of obtained solutions. This may bias the solutions towards reactions not considered in the set of obtained solutions, thus increasing the diversity of the extracted EFMs.

We must avoid weights equal to zero, because this would imply not taking the corresponding reaction into account. Therefore we choose a minimum weight equal to 1 common for all fluxes (reactions). We propose to use as weight for a reaction *r*_*i*_ the number

$$w_{i}=1+p\cdot F_{i}$$

where *p* is called the penalization of the method, and *F*_*i*_ is the frequency of the appearance of the corresponding reaction in the previous set of solutions. Equation 4 shows the new linear program where penalties have been included.

4 $$\begin{array}{*{20}l} \text{Minimize} \quad & \sum\limits_{i=1}^{n} w_{i} \cdot v_{i} \\ \text{subject to} \quad & S \cdot \vec{v} = \vec{0}  \\ & v_{i} \geq 0 \qquad \qquad \;\; \forall r_{i} \in R  \\ \text{where} \quad \quad \;\; & w_{i} = 1+ p \cdot F_{i} \quad \forall r_{i} \in R  \end{array} $$

As it can be deduced, the higher is the frequency *F*_*i*_ the higher *w*_*i*_ becomes and, over a threshold, the optimizer is induced to consider other less penalized reactions (if the structural network properties allow it). The proposed modification of the objective function weights should result in differences between the frequencies of a non-penalized experiment (*F*_*i*_) and the penalized one (*F*^′^_*i*_). Algorithm 3 extends the more general Algorithm 1 by adding the modification of the objective function and the computation of occurrences and frequencies for each reaction in the set of obtained solutions.


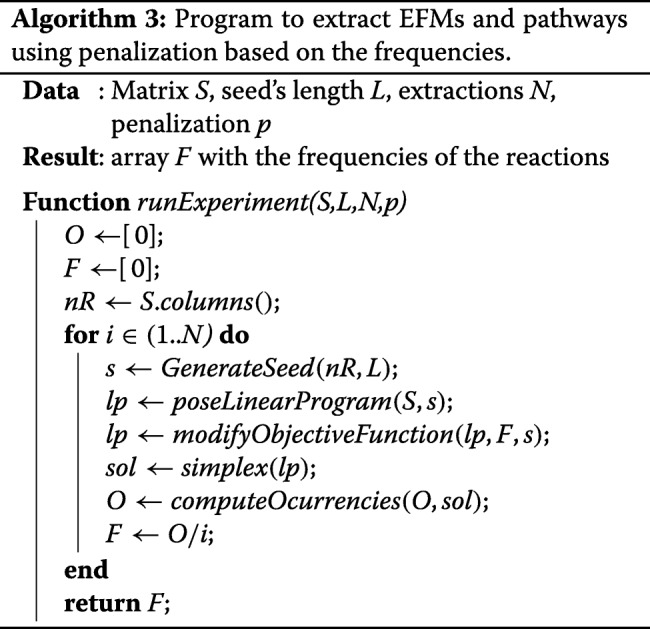


### Comparing set of solutions

Being able to compare different pathway extraction experiments is required in order to measure the effectiveness of our penalization strategy. Differences in the sets of obtained frequencies *F* between penalized and non-penalized experiments would involve that the penalization approach has an impact in the obtained solutions.

Be *F* and *F*^′^ the resulting frequencies in two different experiments, the question is how to measure the possible differences between them. In other words, our objective is to determine whether two samples were selected from populations with the same distribution or not. Observe that we cannot compare these two samples performing a standard *chi-square* test because the values *F*_*i*_ are not independent in general. As we have mentioned before, there are often structural dependencies between reactions and metabolites that can force two related frequencies to be the same. Thus, we have chosen another statistical test, the Wilcoxon signed rank test [26].

To analyse the differences between *F* and *F*^′^ we can use the well-known statistic (denoted by $\overline {\chi }^{2}$) that comes from the *chi-square* test (Eq. 5).

5 $$ \overline{\chi}^{2} = \quad \sum\limits_{i=1}^{n} \frac{{\left({F'}_{i} - F_{i}\right)}^{2}}{F_{i}}   $$

As in the usual *χ*^2^ test, $\overline {\chi }^{2}$ provides a good measure of the differences between the values of *F* and *F*^′^ even if we cannot assure that it corresponds to the *chi-square* distribution. That is, greater values of our statistic means a greater difference between the corresponding experiments but we cannot use it to assign a probability to assure that this difference is statistically significant.

This test measures the differences between frequencies taking into account all the possible factors that could be causing them: the seed generator, the LP solver, the coupling relationships, the size of the GSMN, the number of iterations for the experiment and the penalization. Fixing all the factors except the penalization and choosing a seed generator as neutral as possible, we can study the impact of the penalization in the difference between our non-weighted experiment and the weighted ones. It is expected that higher values of *p* should provide higher values of the statistic which would mean that he behaviour of our extraction method changes.

## Results and discussion

### Configuration of the experiment

The first characteristic we need to tune is the seed generator. The best approach to elucidate the impact of reformulating the objective function is to eliminate any bias produced by the seed generator. For that, we propose to use a uniform random generator because other types of seed generator not based on randomness could introduce certain bias. For example, the adjacency concept used in graph exploration based methods favours the minimality and the feasibility but at the same time harms the independence of selecting reactions to be in a seed.

Then, the seed’s length is also important. The shorter a seed is, the less constrained is the LP and thus it is easier and faster to be solved. Related with the length of the seed but also with the size of the full metabolic network, seeds different enough to each other are required in order to obtain statistically significant differences in the results. According to these considerations, in this study we generate random seeds consisting of sets of 4 reactions.

We have selected two metabolic reconstructions for this study. The first one is *iAF1260*, the reconstruction of the *E. coli K-12 MG1655* organism [27]. The iAF1260 stoichiometric matrix has 3234 de-doubled reactions. The second reconstruction we use is *core E. coli metabolic model* [28]. It is a subset of iAF1260 described as an educational guide with 154 de-doubled reactions.

The first step in our study is to guarantee that the possible differences are in fact related to the inclusion of penalizations. To do so, our start point is to characterize the similarity of the frequency distributions of two experiments using our random pathway extractor without penalization. As a random generator we use the standard Linux one in order to generate seeds consisting in sets of 4 reactions. Every finished experiment provides the frequency of occurrence for each reaction, which is compared between experiments. The experiment consists of several runs of the same number of iterations, the same proportional penalization and the same metabolic reconstruction.

### The influence of the penalizations

As it has been said before, we are interested in knowing if the introduction of penalizations have a significant impact on the results obtained.

Table 1 shows what happens when we introduce penalizations. In this case, each experiment consists of 50.000 iterations. To visualize the effect of the penalization, we have applied the Wilcoxon signed-rank test. The first row shows the result of the test for two experiments without penalization and the following rows show the test for experiments with different penalizations.

**Table 1:** Wilcoxon signed-rank test experiments over core *E. coli* GSMN

Penal. 1	Penal. 2	Wilcoxon *p*-value
0	0	1
0	2	0.0001926
0	5	5.843e-06
2	5	3.262e-07

It is well-known that high Wilcoxon *p*-values indicate a high similarity between the two compared frequencies while low values mean that there are significant differences between the outcomes. Starting with the first row, we can observe that if we ran two experiments without penalizations, the obtained frequencies are (statistically) almost equal. This is important because we can assure that if we find differences between frequencies they are not caused by random oscillations produced by the seed generator.

By observing the first three rows we can assure that the results of experiments with and without penalizations are clearly different and that this difference seems to grow with the value of the penalization. The last row shows that experiments with different penalizations have also a different behaviour. It was clear from rows two and three that there were differences and now we can see that this difference is clearly significant.

Table 2 shows a similar behaviour for the other proposed network. This network is significantly larger than the previous one.

**Table 2:** Wilcoxon signed-rank test experiments over iAF1260 GSMN

Penal. 1	Penal. 2	Wilcoxon *p*-value
0	0	1
0	2	7.507e-05
0	5	0.001265

The experiments has consisted in 50,000 iterations and random seeds of 4 reactions

Therefore, we conclude that the introduction of weights in the objective function has an influence in the results, and that there is also a clear difference between different weights. The above tables also suggest that bigger values of the penalization have more impact in this behaviour.

### Iterations and penalizations

We are also interested in the possible influence of the penalization over the set of frequencies in experiments with a different number of iterations. Table 3 shows the comparison between different experiments for each reconstruction and diverse amount of iterations. A comparison is done applying the *chi-square* based statistic. It shows how the penalization has an influence over the chi-square value considering the same number of iterations.

**Table 3:** Evolution of chi-square test depending on the length of the experiment and the penalization for the core *E. coli* reconstruction

Iterations	Penal. 1	Penal. 2	Chi-square
10K	0	2	0.422773
10K	0	5	0.736042
10K	0	20	1.060799
10K	2	20	0.273936
50K	0	2	0.373511
50K	0	5	0.682528
50K	0	20	1.010308
100K	0	2	0.352029
100K	0	5	0.703740

Table 3 also shows a remarkable impact on the experiment when a penalization is applied. If the number of iterations is fixed and the initial penalization is 0 (see, for example the first three rows of the table), the difference between the frequencies obtained with or without penalizations is clearly increased in function of the value of the penalization.

However, the main part of this difference seems to be achieved with relatively low values of the penalization. If we compare two sets of frequencies obtained with different penalizations the difference between them seems to be less significant (compare, for example, the first and the fourth rows of the table).

It also seems that there is some kind of connection between the length of the experiments and the influence of the penalizations. Experiments with a higher number of iterations should be performed in order to get accurate conclusions.

**Fig. 2:**
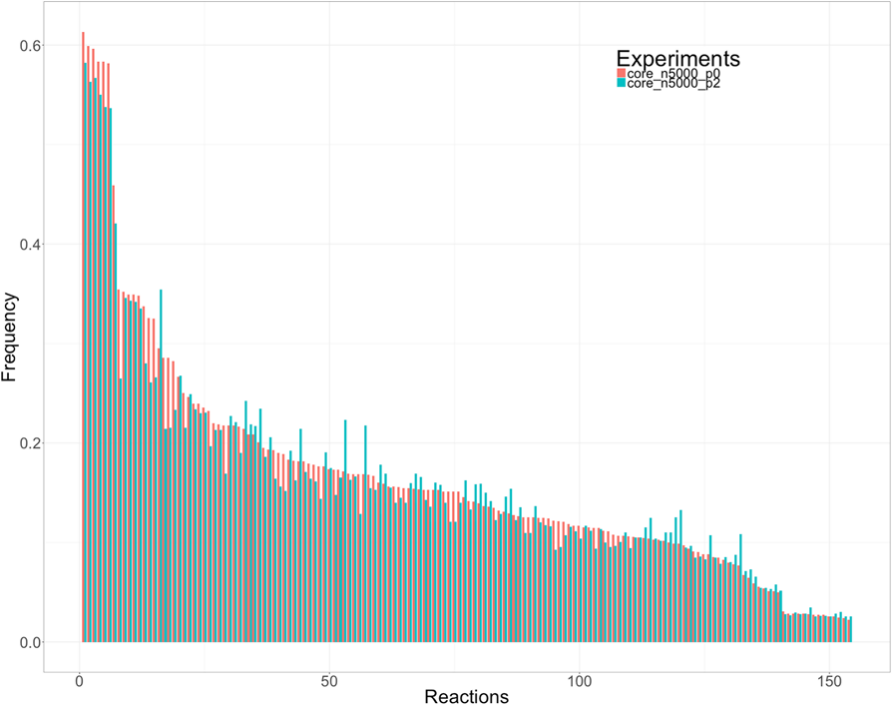
Comparison of two experiments with different penalization. Bar graph grouping the frequencies of two experiments with different penalization (*p* =0 and *p* =2) over *core E. coli metabolic model*. A seed has 4 reactions. The experiments have consisted in 5000 iterations

Once we have checked that using penalizations changes the set of frequencies obtained in one experiment, we try to evaluate these changes among different runs. Figure 2 shows a bar graph where the reactions have been ordered by frequencies in ascendant order. A difference is sensed and it seems to confirm the previous numeric results. It can be also observed that some reactions keep their supremacy over the rest even after trying to reduce its frequency with penalizations.

**Fig. 3:**
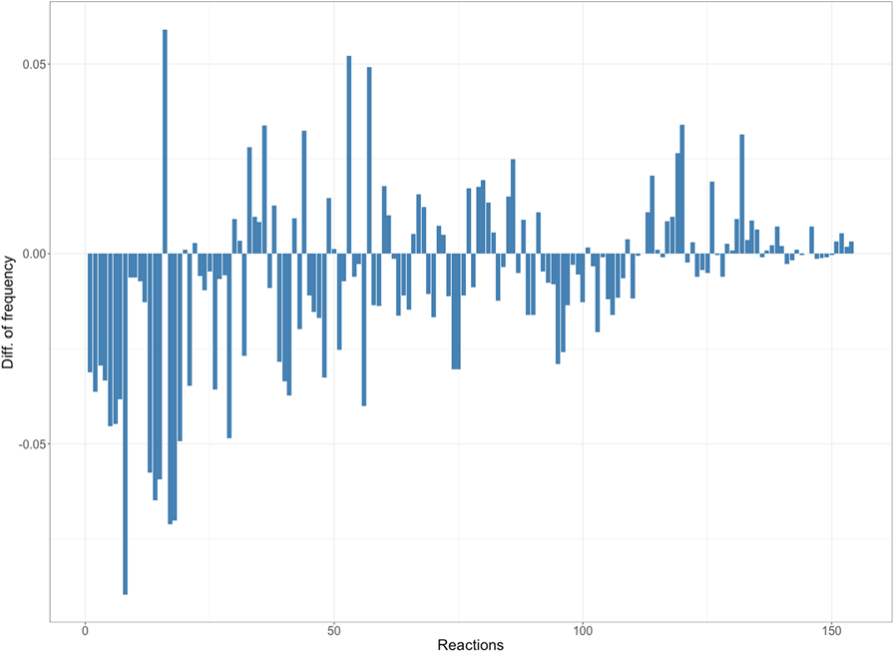
Increment of frequency over an experiment without penalization. Bar graph representing the subtraction of the frequencies of two experiments with different penalization (p =0 and p =2) over *core E. coli metabolic model*. A seed has 4 reactions. The experiments have consisted in 5000 iterations

Moreover, Fig. 3 shows a different view of previous experiments, representing the subtraction of the frequencies of two experiments with different penalization (*p* =0 and *p* =2). Negative bars reflect how some reactions are persuaded to be so frequent, and the positive ones represent that those reactions are being included in the set of solutions more often. The expected result was that the greater is the previous frequency, the most negative is the difference between both experiments. Figure 3 almost let us visualize the expected result but, as commented before, there are other factors within the cell that prevent the solver from ignoring the inclusion of some reactions despite the penalization. More experiments should be done in the future to extract the meaning of the phenomenons like the persistent supremacy of some reactions or the easy variability for some others.

## Conclusions

In this paper, a new EFM extraction strategy is proposed. It can be used together with other LP-based methods. A penalization associated to the previous occurrence of each reaction during an experiment provides information that the LP solver uses to avoid the recurrent appearance of the same reactions in the full set of solutions. As main effect, the solutions are more likely to be diverse. Additionally, rarely included reactions are better represented in the set of solutions. Other factors can affect the effectiveness like the size of the GSMN and the number of iterations of the experiments.

The biological relevance of the extracted EFMs is at least the same that it is supposed to the full set of EFMs. Recently, context-specific approaches are focused on extract a subset of EMFs where a set of reactions are present [22, 23]. Our proposal let implement an intermediate strategy where the not promoted reactions could be penalized.

Regarding future work, it could be relevant to study what results are obtained by doing longer experiments. Besides, frequency distribution and the apparent convergence of the set of solutions using a combination of the proposed factors induces us to think in a measure of “knowledge that holds a extracted subset of pathways from the total remaining”. Furthermore, it seems relevant to us the analysis of the shape of the distribution of solutions and the over and under representation of reactions on them.
